# Social support and community embeddedness protect against post-disaster depression among immigrants: a Vietnamese American case study

**DOI:** 10.3389/fpsyt.2023.1075678

**Published:** 2023-08-31

**Authors:** NhuNgoc K. Pham, Mai Do, Jannette Diep

**Affiliations:** ^1^Department of Social, Behavioral, and Population Sciences, Tulane University School of Public Health and Tropical Medicine, New Orleans, LA, United States; ^2^Department of International Health and Sustainable Development, Tulane University School of Public Health and Tropical Medicine, New Orleans, LA, United States; ^3^BPSOS-Houston, Houston, TX, United States

**Keywords:** disaster, immigrants, health disparities, Hurricane Harvey, Vietnamese American, mental health, social support, depressive symptoms

## Abstract

Immigrants often face increased vulnerabilities to disaster-related poor health and recovery, compared to mainstream populations. Little is known about Hurricane Harvey’s impacts among the storm affected area’s large Vietnamese American population. Our study documented diverse psychological experiences and recovery challenges 1 year post-storm among a sample of 120 Vietnamese Americans residing in the Houston, Texas area. Using linear regression modeling, we examined the association between social support and depressive symptom development among these storm-affected Vietnamese Americans. Social support encapsulating both social embeddedness and perceived support was measured by the Louisville Social Support Scale and depressive symptom development was measured by 18 items that assessed emotional distress. These items included loss of appetite, loss of concentration, exposure to persistent pain, and the exhibition of hopelessness, tiredness, sadness, frustration, discouragement, desperation, exhaustion, disgraced, anger, and craziness. We found adverse post-disaster health outcomes, as well as potential avenues to mitigate them, that should be taken into consideration in the design and implementation of inclusive disaster programs. A high level of social support lowered depressive symptomology among Vietnamese Americans post-Hurricane Harvey, even when accounting for Hurricane Harvey-related home damages and injuries/illness. The negative association between social support and depressive symptom development remained after accounting for both post-storm self-rated mental and physical health. Our results suggested that public health practitioners and emergency management entities should prioritize social support resources to foster mental well-being after hurricanes among Vietnamese Americans as future hurricanes are expected to be stronger and more prevalent along the United States Gulf Coast.

## Introduction

Disaster literature widely documents that individuals exposed to large disasters with cascading effects that evolve overtime, such as hurricanes, are more likely to experience increased levels of stress, anxiety, depression, and post-traumatic stress disorder (PTSD) compared to unexposed individuals (1–9). More so, residents of the United States (U.S.) Gulf Coast often experience elevated levels of depression and anxiety in the immediate aftermath of a hurricane and continue to experience ongoing psychopathologies long after the storm (3, 4). Such psychopathology trends may be present due to Hurricane Harvey, likely the most devastating storm in the last decade. After its landfall on August 25, 2017, the storm destroyed more than 12,000 homes and affected 13 million people across five states: Texas, Louisiana, Mississippi, Tennessee, and Kentucky (10–12). The category 4 hurricane caused widespread flash flooding across most of Harris County, submerging a third of the Houston metropolitan area (13). Existing studies revealed that Texas residents affected by Hurricane Harvey reported poorer self-rated mental health post-storm compared to the national standards (14). Also, individuals who experienced personal or property loss due to the hurricane were more likely to develop mental health symptoms, including PTSD, after the storm (15).

Short- and long-term post-disaster outcomes can differ by individual’s social economic status (SES) and racial backgrounds (16). Older age, occupational status, marital status, acculturation level, and the extent of property damage have statistically significant effects on post-storm health outcomes (17). Poor acculturation among immigrants and extensive property damage negatively affect the outcomes, and they often continue to impede recovery long after the storm (17). Furthermore, immigrants are vulnerable to poor post-disasters recovery due to their limited capacity to prepare for and respond to disasters (18, 19). Often exhibiting low levels of language skills, social acculturation, and SES, immigrants are more prone to poor recovery, and poor physical and mental health outcomes post-disasters (2, 20–30). This is particularly true among Vietnamese Americans across the U.S., who in 2019, reported that 65% of those ages 5 or above experienced limited English proficiency (31).

Previous studies on Hurricane Katrina highlighted that Vietnamese Americans who experienced displacement, severe property loss, and trauma due to the storm experienced concerning prevalence of partial PTSD during the first year post-storm (32). They also had limited access to health care and medications after Hurricane Katrina. About 40% of those surveyed also reported difficulties in obtaining health care needs 1 year post-storm (33, 34). Similar trends may be present among Houston-area Vietnamese American residents affected by Hurricane Harvey, but there have been no data available. However, the safety net provided by high perceived social support and community embeddedness among this community may be helpful in their recovery process; yet such associations among Vietnamese Americans, who are often excluded in post-disaster research, remain underexplored.

Sociodemographic background, pre-disaster health status, event exposure characteristics, and social support factors are features that may be associated with the likelihood of mental health issue development after disasters among affected individuals (35). Existing studies documented the associations between high perceived social support and community embeddedness to better post-disaster outcomes (36–39). Helpful relationships may provide emotional, informational, and instrumental support to alleviate mental distress, reduce anxiety, and enhance feelings of security to help affected individuals navigate the altered landscape of daily life post-disaster (35, 40). For example, social support through social connections may benefit the appraisal process of stressors to help regulate the intensity of emotional reactions to disaster events and subsequently alter post-traumatic stress levels (41–43). The availability of a supportive environment through social relationships and community embeddedness may encourage individuals to express their thoughts and feelings without fears of judgements, fostering the validation of their emotions, to then enhance positive coping and self-efficacy for disaster recovery. The interaction between high self-efficacy and quality social support may further offset negative disaster consequences. Thus, social support may be a critical factor in post-storm recovery to distinguish between those who succumbs to disaster hardships and those who do not (44). More specifically, high perceived social support and social embeddedness – two dimensions of social support – are vital to successful disaster recovery (40, 45–47). Evidence supporting the association between high levels of social support and lower depressive symptoms is expansive within existing literature. However, such association among Vietnamese Americans within the disaster context remain scant, particularly those affected by Hurricane Harvey.

This study is set in the Houston metropolitan area, home to the second largest population of Vietnamese Americans (48). At the time of the study, little was known about the Vietnamese American experiences from Hurricane Harvey in 2017, the second costliest storm to mainland U.S. since 1990 (49). This study thus aims to examine the association between social support and depressive symptom development within a community in the Houston-area that was severely impacted by Hurricane Harvey. We hypothesized that a high level of social support was likely associated with reduced depressive symptom development among Vietnamese Americans post-Hurricane Harvey.

## Materials and methods

### Data collection and sample

In partnership with Boat People SOS (BPSOS)-Houston, a non-profit social and legal service provider, serving primarily first-generation Vietnamese Americans in the area, 120 participants were selected at random from a list of 600 BPSOS-Houston clients. One-on-one interviews were conducted using a structured survey between November 2018 and February 2019, approximately 1 year after the storm. Participant selection criteria were: (1) be a Vietnamese American aged 18 or older; (2) have experienced Hurricane Harvey; and (3) received direct or indirect assistance related to the hurricane through BPSOS-Houston. Study participants are not representative of all Vietnamese Americans in Houston-area, but of those affected residents seeking assistance post-storm at BPSOS-Houston. The questionnaire cross-sectionally asked questions about depressive symptoms experienced after the storm, perceptions of social support and embeddedness, self-rated physical and mental health after the storm, and hurricane related damages and losses. We also collected information on socio-demographic characteristics, English language skills and language preference, healthcare access, and pre-storm characteristics among participants, including employment status and residency. Interviewers, trained by the Principal Investigator in ethical conduct, protection of participants’ privacy and confidentiality, and interview skills, conducted data collection in English or Vietnamese, based on participants’ preference. The Tulane University’s Institutional Review Board provided approval for this study.

### Variables

Our outcome of interest is depressive symptoms captured by the Vietnamese Depression Scale (50, 51). The Vietnamese Depression Scale was originally developed for Vietnamese refugees in the U.S. by Kinzie and colleagues (51) in 1982 to assess depression with somatic symptoms prevalent among Asian populations. Validity testing of the Vietnamese Depression Scale demonstrated that it appears to be a psychologically sound tool for screening depressive symptomatology among community dwelling Vietnamese refugees in Houston, Texas (50). The scale included 18 items that assessed emotional distress within the past 7 days of the interview. These items included loss of appetite, loss of concentration, exposure to persistent pain, and the exhibition of hopelessness, tiredness, sadness, frustration, discouragement, desperation, exhaustion, disgraced, anger, and craziness. The scale demonstrated excellent internal consistency (Cronbach’s alpha = 0.90) among this sample. A sum of symptoms present, ranging from zero (lowest) to 18 (highest), created the overall depressive symptom score. Existing literature proposed two Vietnamese Depression Scale cut-off points for the presence of major depression: ≥12 symptoms (52) and ≥13 symptoms (53). The first study tested the cut-off point among newly arrived (i.e., ≤6 months) Vietnamese refugees to the U.S. using a modified 15-item Vietnamese Depression Scale (52). We decided against using the cut-off points to not lose detailed information applicable to our study. We used the overall depressive symptom score in its continuous forms in our analysis.

Our key independent variable is social support measured by the Louisville Social Support Scale (LSSS) (54–56). The LSSS measures the extent and closeness of one’s social network and the overall degree of satisfaction with support provided by that network overtime. Respondents were asked to reflect on the social support network in totality before providing an average rating of the support provided by the network. The 13 items within the LSSS asked about how frequently participants saw family, friends, or neighbors; the extent of involvement in community organizations; and how much help they could expect to receive from family, friends, or neighbors during an emergency. The LSSS centers on two subscales, social integration or embeddedness within a social network (i.e., social embeddedness), and expected support or help. Expected support assesses specific expectations of help in an emergency from family, friends, and the community; therefore, measures perceived social support (54). All responses were categorical, and the number of responses category for each question ranged from three to five; thus, it was necessary to standardize all the items first before creating an averaged scale. The scale demonstrated very good internal consistency (Cronbach’s alpha = 0.80) among our sample. We retained the LSSS score in its continuous form in our analysis. A higher score indicated higher perceived social support and social embeddedness; thus, higher overall social support.

Other key independent variables were those related to Hurricane Harvey, including: (1) damaged home and (2) any sustained injury or illness from the storm; both were binary. We also measured post-disaster self-rated physical health and mental health by component scores of the Medical Outcomes Study Short-Form-12 (SF-12), version 1 (57, 58). We used the Vietnamese version of the SF-12 that was constructed, pretested, and implemented in other larger studies among Vietnamese Americans (16, 17, 59). The SF-12, widely used to assess self-rated mental and physical functioning, includes eight indicators: physical function; role physical; bodily pain; general health; vitality; social functioning; role emotional; and mental health. Indicators used in the mental component score (MCS) development are vitality, social functioning, role emotional, and mental health while indicators used in the physical component score (PCS) development are physical function, role physical, bodily pain, and general health (58). We derived the MCS and PCS, ranging from zero (lowest) to 100 (highest), from the eight indicators described above in accordance with the methods outlined by the scale authors, Ware et al. (58, 60). Higher scores indicated better mental or physical health status (60, 61).

We used binary coding of no (0) and yes (1) to distinguish healthcare access and other pre-storm characteristics: possession of health insurance; routine physical exam within the last 12 months; employment status before the hurricane; and having lived at the same address for five or more years. Individual socio-demographic characteristics collected included: age; sex; monthly income; marital status; education level; religion affiliation; and acculturation level. We coded all basic socio-demographic characteristics, except for religious affiliation, as binary variables of 0 and 1: age (<65 years or ≥65 years of age), sex (female or male), monthly income level (low, i.e., <$1,500 of median income or high, i.e., ≥$1,500), marital status (married/living with spouse/partner or never married or divorced/widowed), and highest education level (≤12 grade or >12 grade). We selected the age cut-off at 65 to distinguish older adults versus those of working age, while $1,500 was the median cut-off of the sample’s income. We categorized religious affiliations between three classifications: Catholic; Buddhist; and no religions/others. We determined acculturation levels based on language fluency and cultural preference using the Anderson’s Acculturation Scale for Southeast Asian immigrants (62), then grouped the levels as either low (0) or high (1) using the median acculturation factor analysis score reported among the sample. The 17 items we used to construct the acculturation scale demonstrated very good internal consistency (Cronbach’s alpha = 0.86). Higher scores indicate higher levels of assimilation to the U.S. society and better command of the English language.

### Analysis

We calculated descriptive statistics with bivariate and multivariable analyses using Stata17 (63). We used linear regression modeling to assess the association between social support and depressive symptom score while controlling for Hurricane Harvey-related variables and socio-demographic characteristics in Model 1 through Model 3. The goal of these analyses was to determine whether and how social support affected post-storm depression. To further distinguish the effect of social support on depressive symptoms regardless of post-storm health status, we controlled for self-rated MCS and PCS within Model 4. We reported the beta coefficient (*b*) and standard error (S.E.) for each variable; a value of *p* of less than 0.05 indicated statistical significance.

## Results

The mean depressive symptom score among participants was 5.94 (standard deviation [SD] = 4.84); more specifically, more than half of participants (52.50%) experienced five or more symptoms – the median number of symptoms – 1 year post-storm (not shown in Table 1). The mean SF-12 MCS was 32.63 (S.D. = 4.98) while the mean SF-12 PCS was 39.78 (S.D. = 10.15). Although 75.83% of participants experienced storm-related home damages, less than a quarter of participants experienced injuries or illnesses due to the storm (Table 1). The mean age of participants was 57 and 71.43% were below the age of 65. The distributions of sex and education level among participants were relatively even (Table 2). The mean monthly income among participants was $1,903 (not shown in Table 2) while the median monthly income was $1,500. Prior to Hurricane Harvey, 66.67% of participants had employment and the majority (70.00%) had consistent residency pre- and post-storm. The percentage of uninsured participants was high (34.17%) compared to the national percentage of uninsured Vietnamese Americans. Most participants (69.17%) had a routine physical exam within the last 12 months prior to the interview. About half of participants indicated a high level of acculturation.

**Table 1:** Distribution of post-disaster outcomes and Hurricane Harvey-relatedcharacteristics among study sample of Vietnamese Americans (*N* = 120) in Houston, 2018–2019.

Variables	Mean or %	S.D.	Min.	Max.
Post-Disaster Outcomes
Depressive Symptom Score	5.94	(4.84)	0	18
Social Support Score (LSSS^a^)	−0.00	(0.55)	−1.02176	1.332634
Mental Health Score Post-Storm (SF-12 MCS^b^)	32.63	(4.98)	19.47309	45.63055
Physical Health Score Post-Storm (SF-12 PCS^b^)	39.78	(10.15)	17.55211	58.26475
Hurricane-Harvey Related Characteristics
Experienced Damaged Home Due To Storm				
No	24.17%			
Yes	75.83%			
Sustained Injury/Illness Due To Storm				
No	75.83%			
Yes	24.17%			

a Louisville Social Support Scale *z*-score transformed.

b Derived from SF-12 composite scores.

S.D., standard deviation; Min, minimum; Max, maximum.

**Table 2:** Distribution of socio-demographic characteristics among study sample of Vietnamese Americans (*N* = 120) in Houston, 2018–2019.

Variables	Percentage
Age
<65	71.43%
≥65	28.57%
Sex
Female	53.33%
Male	46.67%
Monthly income (Median = $1,500)
<$1,500	41.67%
≥$1,500	58.33%
Health insurance
No	34.17%
Yes	65.83%
Married/Partnered
No	36.67%
Yes	63.33%
Education level
≤12 grade	54.17%
>12 grade	45.83%
Religion
Catholic	47.50%
Buddhist	40.83%
No religion/others	11.67%
Employed pre-hurricane Harvey
No	33.33%
Yes	66.67%
Living at the same address for ≥5 years
No	30.00%
Yes	70.00%
Had routine physical exam within last 12 months
No	30.83%
Yes	69.17%
Acculturation level
Low	49.17%
High	50.83%

Within Table 3, we presented the results to our models that examined the predictors of depressive symptom scores among the participants. Model 1 included only social support as measured by the LSSS to predict depressive symptom scores. We added socio-demographic characteristics in Model 2 to ensure that the observed association between social support and depressive symptom development was not confounded by factors such as income and acculturation, while Model 3 included Hurricane Harvey-related characteristics. In Model 4, we added SF-12 MCS and SF-12 PCS measures to assess if the observed association between social support and depressive symptomology was confounded by self-rated mental health and/or physical health after the hurricane.

**Table 3:** Multivariable linear regression models for the effects of social support, Hurricane Harvey-related characteristics, and other covariates on depressive symptom score among study sample of Vietnamese Americans (*N* = 120) in Houston, 2018–2019.

Variables	Model 1		Model 2		Model 3		Model 4	
	Coeff.	S.E.	Coeff.	S.E.	Coeff.	S.E.	Coeff.	S.E.
Social Support Score (LSSS^a^)	−1.91*	(0.81)	−2.31**	(0.85)	−2.34**	(0.80)	−1.80*	(0.76)
≥65 Years Of Age			0.38	(1.23)	0.26	(1.15)	−0.32	(1.13)
Male			−0.38	(0.92)	−0.77	(0.87)	−0.54	(0.87)
Income Level ≥ $1,500			−0.13	(0.98)	−0.04	(0.92)	−0.27	(0.89)
Insured			−1.42	(0.93)	−1.15	(0.88)	−0.80	(0.84)
Married/Partnered			0.31	(1.04)	0.41	(0.97)	0.77	(1.01)
Education Level > 12 Grade			−2.04*	(0.93)	−1.91*	(0.87)	−0.82	(0.88)
Religion
Catholic			0.00	(0.00)	0.00	(0.00)	0.00	(0.00)
Buddhist			−1.44	(1.00)	−1.41	(0.94)	−1.32	(0.94)
No Religion/Others			−1.16	(1.47)	−1.20	(1.38)	−2.20	(1.32)
Employed Pre-Storm			−2.46*	(1.17)	−2.07	(1.11)	−0.40	(1.08)
Living At The Same Address For ≥5 Years			−1.50	(0.97)	−1.26	(0.91)	−0.83	(0.87)
Had Routine Physical Exam Within Last 12 Months			−0.16	(1.04)	0.11	(0.98)	0.17	(0.97)
Has High Acculturation Level			−0.70	(0.89)	−1.09	(0.84)	−1.20	(0.83)
Experienced Damaged Home Due To Storm					−0.21	(1.01)	−0.40	(1.04)
Sustained Injury/Ill Due To Storm					3.90***	(0.97)	1.88	(1.05)
Mental Health Score Post-Storm (SF-12 MCS^b^)							−0.13	(0.09)
Physical Health Score Post-Storm (SF-12 PCS^b^)							−0.24***	(0.05)
Intercept
Number Of Observations	118.00		117.00		117.00		103.00	
F Statistic	5.62		2.21		3.28		4.64	
R-Squared	0.05		0.22		0.33		0.48	
Log Likelihood	−350.65		−336.36		−327.53		−275.89	

a Louisville Social Support Scale *z*-score transformed.

b Derived from SF-12 composite scores.

***p < 0.001, **p < 0.01, and *p < 0.05. Coeff, coefficient; S.E., standard error.

Social support, consistently across all four models, had a negative association with the outcome, in which higher social support was associated with lower depressive symptom scores (*p* < 0.05). Higher education was significantly related to lower depressive symptom scores in both Model 2 and Model 3 (*p* < 0.05); it seemed protective against depression symptom development in Model 4 but without statistical significance (*b* = −0.82, *p* > 0.05). Pre-storm employment had a negative association with depressive symptom score in Model 2 (*b* = −2.46, *p* < 0.05) but became statistically insignificant in Model 3 and Model 4.

Those who sustained injuries or illnesses from the storm experienced almost four points higher depressive symptom scores compared to those that did not in Model 3 (*b* = 3.90, *p* < 0.01). However, storm injuries or illnesses lost its statistical significance in Model 4. High self-rated physical health (*b* = −0.24, *p* < 0.001) was a statistically significant predictor of depressive symptom score, in which higher physical health was associated with lower depressive symptom scores in Model 4. The results implied that the influence of storm-related injuries or illnesses on depressive symptom development following Hurricane Harvey was explained in part by the participants’ perception of overall physical health 1 year post-storm; therefore, sustaining injuries or illnesses from the storm rendered marginally statistical significance (*p* = 0.08) in Model 4.

Residency tenure and high acculturation level consistently showed negative, but statistically insignificant, associations with depressive symptom scores across models. Individual characteristics, including age, sex, income level, health insurance status, marital status, and religion were not associated with the outcome in any models.

We conducted multicollinearity assessments in Model 4 using the variance inflation factor (VIF) metric to examine the correlation and strength of correlation between the predictor variables in the models. None of the VIF values were high, while the mean VIF value was 1.39, which indicated that multicollinearity was not a concern. In addition, a correlation matrix revealed that predictor variables were not highly correlated to one another (not shown).

## Discussion

Our study results documented the protective impact that social support has on depressive symptom development post-Hurricane Harvey. The results confirmed our hypothesis that a high presence of social support lowers depressive symptom scores among Vietnamese Americans post-Hurricane Harvey, even when accounting for Hurricane Harvey-related home damages and storm-related injury or illness. More so, the negative association between social support and depressive symptom development remained when accounting for both post-storm self-rated mental and physical health.

Better physical health was associated with fewer depressive symptoms. High acculturation level indicated better assimilation into U.S. society and better knowledge of the English language which may be helpful for Vietnamese Americans to navigate recovery resource access; thus, mitigating post-storm distress. However, in our study, although high acculturation level decreased depressive symptom development, the association lacked statistical significance (*b* = −1.19, *p* > 0.05). Inconsistent with existing studies, marriage/partnership did not have a statistically significant protective relationship to psychological distress development post-storm (4, 6, 36, 64). Interestingly, despite statistical insignificance, home damages due to the storm did not elevate depressive symptoms among our participants like it did for Vietnamese Americans affected by Hurricane Katrina (32).

Our results emphasized that Vietnamese Americans could be vulnerable to the adverse impacts of natural disasters long after the storm. More than 1 year post-storm, depressive symptoms remained prevalent among participants. Prolonged depressive symptoms may contribute to negative long-term well-being and health outcomes among Vietnamese Americans impacted by Hurricane Harvey. Disaster-related pessimistic thoughts are linked to physical health problems, more specifically, existing studies revealed that enduring negative thoughts contribute to anxiety disorders and chronic illnesses such as cardiovascular diseases over long durations (65, 66). Thus, prolonged psychological distress may result in poor recovery to pre-storm well-being and health among Vietnamese Americans affected by the storm (44, 67). The implication is that negative thoughts post-disaster can be part of a pathway to poorer health and subsequently group health disparities. Our results also indicated that on average, Vietnamese Americans had lower self-rated mental and physical health outcomes post-storm compared to the national standards of 50 (SD = 10). The stress from rebuilding after the storm may have cascading health impacts among this subpopulation but unexplored in existing literature. Such results emphasize that Vietnamese Americans’ well-being, especially psychological distress, immediately and long-term post-storm should be considered in recovery efforts.

Our study was limited by a small sample size and participant recruitment from a list of only BPSOS-Houston clients, even though the selection was random. Thus, the sample may not necessarily be representative of all Vietnamese Americans in the area, some of whom might be lucky enough to not experience Hurricane Harvey. In addition, the cross-sectional data did not allow us to assess depressive symptoms and the health status of participants prior to the storm, which limited causal references regarding the storm’s impacts. Future research using larger samples and replication to Vietnamese Americans outside of the BPSOS-Houston network would build on the strengths of our study. Also, continued long-term follow-up using a longitudinal approach would be beneficial for better understanding of how social support is associated with post-storm psychological distress across time.

## Conclusion

Overall, our study added to the limited literature on post-disaster outcomes and the positive impacts of social support among immigrants and within the Vietnamese Houstonian population. Despite cross-sectional data limitations, our study findings have significant policy implications as the U.S. Gulf Coast continues to experience increased prevalence of powerful hurricanes (68, 69). Our results suggest that mental well-being after hurricanes among Vietnamese Americans should be prioritized by public health practitioners and emergency management entities. Programs and services should focus on fostering mental well-being and mitigating psychological distress through the provision of social support and community cohesion resources. Better efforts to integrate Vietnamese Americans into the larger Houston-area community pre-storms would contribute to building social embeddedness which may be beneficial in preparation for additional future hurricane exposures. As future hurricanes are expected to be stronger and more prevalent, inclusive preparedness efforts are critical. Other immigrant groups with similar characteristics as Vietnamese Americans may share similar disaster vulnerabilities highlighted by our study.

## Data availability statement

The datasets presented in this article are not readily available because protection of participants’ privacy. Requests to access the datasets should be directed to MD, mdo@tulane.edu.

## Ethics statement

The studies involving human participants were reviewed and approved by the Institutional Review Board of Tulane University. The patients/participants provided their written informed consent to participate in this study.

## Author contributions

NP: conceptualization, formal analysis, methodology, software, writing – original draft, writing – review and editing, and visualization. MD: conceptualization, data curation, funding acquisition, methodology, project administrations, supervision, and writing – review and editing. JD: project administrations and writing – review and editing. All authors contributed to the article and approved the submitted version.

## Funding

This project was funded by the Tulane University’s Carol Lavin Bernick Faculty Grant and the Tulane Japan Friendship Award with MDo, MPH, MD, PH as Principal Investigator.

## Conflict of interest

The authors declare that the research was conducted in the absence of any commercial or financial relationships that could be construed as a potential conflict of interest.

## Publisher’s note

All claims expressed in this article are solely those of the authors and do not necessarily represent those of their affiliated organizations, or those of the publisher, the editors and the reviewers. Any product that may be evaluated in this article, or claim that may be made by its manufacturer, is not guaranteed or endorsed by the publisher.

## References

[1] Briere J Elliott D. Prevalence, characteristics, and long-term sequelae of natural disaster exposure in the general population. J Trauma Stress. (2000) 13:661–79. doi: 10.1023/A:1007814301369, PMID: 11109238

[2] Goldmann E Galea S. Mental health consequences of disasters. Annu Rev Public Health. (2014) 35:169–83. doi: 10.1146/annurev-publhealth-032013-182435 24159920

[3] Weisler RH Barbee JG Townsend MH. Mental health and recovery in the Gulf coast after hurricanes Katrina and Rita. JAMA. (2006) 296:585–8. doi: 10.1001/jama.296.5.585, PMID: 16882968

[4] Paxson C Fussell E Rhodes J Waters M. Five years later: recovery from post traumatic stress and psychological distress among low-income mothers affected by hurricane Katrina. Soc Sci Med. (2012) 74:150–7. doi: 10.1016/j.socscimed.2011.10.004, PMID: 22137245 PMC3286602

[5] Rhodes J Chan C Paxson C Rouse CE Waters M Fussell E. The impact of hurricane Katrina on the mental and physical health of low-income parents in New Orleans. Am J Orthopsychiatry. (2010) 80:237–47. doi: 10.1111/j.1939-0025.2010.01027.x, PMID: 20553517 PMC3276074

[6] Raker EJ Lowe SR Arcaya MC Johnson ST Rhodes J Waters MC. Twelve years later: the long-term mental health consequences of hurricane Katrina. Soc Sci Med. (2019) 242:112610. doi: 10.1016/j.socscimed.2019.112610, PMID: 31677480 PMC8450020

[7] Schwartz RM Sison C Kerath SM Murphy L Breil T Sikavi D. The impact of hurricane Sandy on the mental health of New York area residents. Am J Disaster Med. (2015) 10:339–46. doi: 10.5055/ajdm.2015.0216 27149315

[8] Neria Y Nandi A Galea S. Post-traumatic stress disorder following disasters: a systematic review. Psychol Med. (2008) 38:467–80. doi: 10.1017/S0033291707001353, PMID: 17803838 PMC4877688

[9] Guilaran J de Terte I Kaniasty K Stephens C. Psychological outcomes in disaster responders: a systematic review and meta-analysis on the effect of social support. Int J Disaster Risk Sci. (2018) 9:344–58. doi: 10.1007/s13753-018-0184-7

[10] Moravec ER. Texas officials: hurricane Harvey death toll at 82, ‘mass casualties have absolutely not happened.’. Wash Post. (2017). Available at: https://www.washingtonpost.com/national/texas-officials-hurricane-harvey-death-toll-at-82-mass-casualties-have-absolutely-not-happened/2017/09/14/bff3ffea-9975-11e7-87fc-c3f7ee4035c9_story.html

[11] Historic Disaster Response to Hurricane Harvey in Texas [press release]. (Accessed September 22, 2017)

[12] Brasier R Thompson J. FEMA to play long-term role in recovery from Harvey. Southwest Economy. (2018, 2018):15–7.

[13] Flores AB Collins TW Grineski SE Chakraborty J. Disparities in health effects and access to health care among Houston area residents after hurricane Harvey. Public Health Rep. (2020) 135:511–23. doi: 10.1177/0033354920930133, PMID: 32539542 PMC7383766

[14] Karaye IM Ross AD Perez-Patron M Thompson C Taylor N Horney JA. Factors associated with self-reported mental health of residents exposed to hurricane Harvey. Prog Disaster Sci. (2019) 2:100016. doi: 10.1016/j.pdisas.2019.100016

[15] Schwartz RM Tuminello S Kerath SM Rios J Lieberman-Cribbin W Taioli E. Preliminary assessment of hurricane Harvey exposures and mental health impact. Int J Environ Res Public Health. (2018) 15:50974. doi: 10.3390/ijerph15050974, PMID: 29757262 PMC5982013

[16] Zhang M VanLandingham M Park YS Anglewicz P Abramson DM. Differences in post-disaster mental health among Vietnamese and African Americans living in adjacent urban communities flooded by Katrina. PLoS One. (2021) 16:255303. doi: 10.1371/journal.pone.0255303 PMC8386886 34432809

[17] Vu L Vanlandingham MJ. Physical and mental health consequences of Katrina on Vietnamese immigrants in New Orleans: a pre- and post-disaster assessment. J Immigr Minor Health. (2011) 14:386–94. doi: 10.1007/s10903-011-9504-3 21789559

[18] Lindell MK Perry RW. Communicating environmental risk in multiethnic communities Sage Publications (2003). doi: 10.4135/9781452229188

[19] Johnson KR. Hurricane Katrina: lessons about immigrants in the administrative state. Hous L Rev. (2008) 45:11.

[20] Kaplan AS Huynh UK. Working with Vietnamese Americans in disasters. Ethnocultural Perspect Disaster Trauma. (2008) 2008:321–49. doi: 10.1007/978-0-387-73285-5_11

[21] Seidenberg J. Cultural competency in disaster recovery: lessons learned from the hurricane Katrina experience for better serving marginalized communities. CA: University of California, Berkeley School of Law (2005).

[22] Shiu-Thornton S Balabis J Senturia K Tamayo A Oberle M. Disaster preparedness for limited English proficient communities: medical interpreters as cultural brokers and gatekeepers. Public Health Rep. (2007) 122:466–71. doi: 10.1177/003335490712200407, PMID: 17639649 PMC1888520

[23] Mathew A Kelly K. Disaster preparedness in urban immigrant communities. A Tomás Rivera Policy Institute and Asian Pacific American Legal Center Report, (2008), 4:2008. doi: 10.1108/dpm.2009.07318bab.002

[24] Nguyen MT Salvesen D. Disaster recovery among multiethnic immigrants: a case study of southeast Asians in bayou la Batre (AL) after hurricane Katrina. J Am Plan Assoc. (2014) 80:385–96. doi: 10.1080/01944363.2014.986497

[25] Norris FH Perilla JL Riad JK Kaniasty K Lavizzo EA. Stability and change in stress, resources, and psychological distress following natural disaster: findings from hurricane Andrew. Anxiety Stress Coping. (1999) 12:363–96. doi: 10.1080/10615809908249317, PMID: 21777067

[26] Collins TW Jimenez AM Grineski SE. Hispanic health disparities after a flood disaster: results of a population-based survey of individuals experiencing home site damage in El Paso (Texas, USA). J Immigr Minor Health. (2013) 15:415–26. doi: 10.1007/s10903-012-9626-2, PMID: 22527746

[27] Elliott JR Pais J. Race, class, and hurricane Katrina: social differences in human responses to disaster. Soc Sci Res. (2006) 35:295–321. doi: 10.1016/j.ssresearch.2006.02.003

[28] Toldson IA Ray K Hatcher SS Louis LS. Examining the long-term racial disparities in health and economic conditions among hurricane Katrina survivors: policy implications for Gulf Coast recovery. J Black Stud. (2011) 42:360–78. doi: 10.1177/0021934710372893, PMID: 21905324

[29] Fergusson DM Horwood LJ Boden JM Mulder RT. Impact of a major disaster on the mental health of a well-studied cohort. JAMA Psychiat. (2014) 71:1025–31. doi: 10.1001/jamapsychiatry.2014.652, PMID: 25028897

[30] Perilla JL Norris FH Lavizzo EA. Ethnicity, culture, and disaster response: identifying and explaining ethnic differences in PTSD six months after hurricane Andrew. J Soc Clin Psychol. (2002) 21:20–45. doi: 10.1521/jscp.21.1.20.22404

[31] https://www.migrationpolicy.org/article/vietnamese-immigrants-united-states.

[32] Norris FH Vanlandingham MJ Vu L. PTSD in Vietnamese Americans following hurricane Katrina: prevalence, patterns, and predictors. J Trauma Stress. (2009) 22:91–101. doi: 10.1002/jts.20389, PMID: 19235888 PMC2923021

[33] Do M Hutchinson P Mai K Vanlandingham M. Disparities in health care among Vietnamese new Orleanians and the impacts of hurricane Katrina. Res Sociol Health Care. (2009) 27:301–19. doi: 10.1108/S0275-4959(2009)0000027016, PMID: 20725605 PMC2923394

[34] Vu L VanLandingham MJ Do M Bankston CL. Evacuation and return of Vietnamese new Orleanians affected by hurricane Katrina. Organ Environ. (2009) 22:422–36. doi: 10.1177/1086026609347187, PMID: 20871780 PMC2943234

[35] Bonanno GA Brewin CR Kaniasty K Greca AML. Weighing the costs of disaster: consequences, risks, and resilience in individuals, families, and communities. Psychol Sci Public Interest. (2010) 11:1–49. doi: 10.1177/1529100610387086, PMID: 26168411

[36] Bui BKH Anglewicz P VanLandingham MJ. The impact of early social support on subsequent health recovery after a major disaster: a longitudinal analysis. SSM Popul Health. (2021) 14:100779. doi: 10.1016/j.ssmph.2021.100779, PMID: 33869723 PMC8040331

[37] Merdjanoff AA Bui B Pendley SC Ballard Z Galliford E VanLandingham M, (Eds.) Drivers of delayed return migration among hurricane Katrina survivors: a mixed-methods analysis. Natural Hazards Workshop, (2021), July 14–15, 2021; On-line for 2021

[38] Norris F Kaniasty K. Received and perceived social support in times of stress: a test of the social support deterioration deterrence model. J Pers Soc Psychol. (1996) 71:498–511. doi: 10.1037/0022-3514.71.3.498, PMID: 8831159

[39] Smiley KT Clay LA Ross AD Chen Y-A. Multi-scalar and multi-dimensional conceptions of social capital and mental health impacts after disaster: the case of hurricane Harvey. Disasters. 46:473. doi: 10.1111/disa.12474 33432691

[40] Kaniasty K Norris FH. Distinctions that matter: received social support, perceived social support, and social embeddedness after disasters. Norris FH Galea S Neria Y, (Eds.) Mental health and disasters. Cambridge: Cambridge University Press; (2009). 175–200

[41] Dai W Chen L Tan H Wang J Lai Z Kaminga AC. Association between social support and recovery from post-traumatic stress disorder after flood: a 13–14 year follow-up study in Hunan, China. BMC Public Health. (2016) 16:194. doi: 10.1186/s12889-016-2871-x, PMID: 26924178 PMC4770534

[42] Harandi TF Taghinasab MM Nayeri TD. The correlation of social support with mental health: a meta-analysis. Electron Physician. (2017) 9:5212–22. doi: 10.19082/5212, PMID: 29038699 PMC5633215

[43] Başoğlu M Kiliç C Salcioğlu E Livanou M. Prevalence of posttraumatic stress disorder and comorbid depression in earthquake survivors in Turkey: an epidemiological study. J Trauma Stress. (2004) 17:133–41. doi: 10.1023/B:JOTS.0000022619.31615.e8, PMID: 15141786

[44] Abramson DM Stehling-Ariza T Park YS Walsh L Culp D. Measuring individual disaster recovery: a socioecological framework. Disaster Med Public Health Prep. (2010) 4:S46–54. doi: 10.1001/dmp.2010.14, PMID: 23105035

[45] Barrera M. Distinctions between social support concepts, measures, and models. Am J Community Psychol. (1986) 14:413–45. doi: 10.1007/BF00922627

[46] Hupcey JE. Clarifying the social support theory-research linkage. J Adv Nurs. (1998) 27:1231–41. doi: 10.1046/j.1365-2648.1998.01231.x, PMID: 9663875

[47] Baptista N Alves H Pinho JC. Uncovering the use of the social support concept in social marketing interventions for health. J Nonprofit Publ Sect Market. (2022) 34:1–35. doi: 10.1080/10495142.2020.1760999

[48] Capps R Soto AGR. A profile of Houston’s diverse immigration population in a rapidly changing policy Lanscape. (2018). Available at: https://www.migrationpolicy.org/research/profile-houston-immigrant-population-changing-policy-landscape

[49] Blake ES Zelinsky DA. National Hurricane Center tropical cyclone report: hurricane Harvey. Administration NOaA, (2018). 77. Available from: https://www.nhc.noaa.gov/data/tcr/AL092017_Harvey.pdf

[50] Dinh TQ Yamada AM Yee BWK. A culturally relevant conceptualization of depression: an empirical examination of the factorial structure of the Vietnamese depression scale. Int J Soc Psychiatry. (2009) 55:496–505. doi: 10.1177/0020764008091675, PMID: 19592442

[51] Kinzie JD Manson SM Vinh DT Tolan NT Anh B Pho TN. Development and validation of a Vietnamese-language depression rating scale. Am J Psychiatry. (1982) 139:1276–81. doi: 10.1176/ajp.139.10.1276, PMID: 7124979

[52] Hinton WL Du N Chen YC Tran CG Newman TB Lu FG. Screening for major depression in Vietnamese refugees: a validation and comparison of two instruments in a health screening population. J Gen Intern Med. (1994) 9:202–6. doi: 10.1007/BF02600124, PMID: 8014725

[53] Murphy J Goldner EM Goldsmith CH Oanh PT Zhu W Corbett KK. Selection of depression measures for use among Vietnamese populations in primary care settings: a scoping review. Int J Ment Health Syst. (2015) 9:31. doi: 10.1186/s13033-015-0024-8, PMID: 26300962 PMC4543473

[54] Stroebe W Stroebe M Abakoumkin G Schut H. The role of loneliness and social support in adjustment to loss: a test of attachment versus stress theory. J Pers Soc Psychol. (1996) 70:1241–9. doi: 10.1037/0022-3514.70.6.1241, PMID: 8667164

[55] Norris FH Murrell SA. Older adult family stress and adaptation before and after bereavement. J Gerontol. (1987) 42:606–12. doi: 10.1093/geronj/42.6.606 3680879

[56] Murrell S Himmelfarb S Schulte P Norris F. Pretest of candidate measures: results and final decisions. Louisville: University of Louisville Urban (1981).

[57] Hays RD Sherbourne CD Mazel R. User's manual for the medical outcomes study (MOS) Core measures of health-related quality of life. Santa Monica, CA: RAND Corporation (1995).

[58] Ware J Kosinski M Keller SD. A 12-item short-form health survey: construction of scales and preliminary tests of reliability and validity. Med Care. (1996) 34:220–33. doi: 10.1097/00005650-199603000-00003 8628042

[59] Fu H VanLandingham MJ. Mental health consequences of international migration for Vietnamese Americans and the mediating effects of physical health and social networks: results from a natural experiment approach. Demography. (2012) 49:393–424. doi: 10.1007/s13524-011-0088-2, PMID: 22275002

[60] Bruun NH. SF12: Stata module. Statistical software components: Boston College Department of economics; 2015. p. SF12: Stata module to validate SF input and calculate SF version 2 t scores

[61] 12-Item short form survey (SF-12), Santa Monica, CA: RAND Coporation; (2022), Available at: https://www.rand.org/health-care/surveys_tools/mos/12-item-short-form.html

[62] Anderson J Moeschberger M Chen MS Kunn P Wewers ME Guthrie R. An acculturation scale for southeast Asians. Soc Psychiatry Psychiatr Epidemiol. (1993) 28:134–41. doi: 10.1007/BF00801744, PMID: 8378809

[63] StataCorp. Stata statistical software: Release 17. 17th ed. College Station, TX: StataCorp LLC (2022).

[64] Chan CS Lowe SR Weber E Rhodes JE. The contribution of pre-and postdisaster social support to short-and long-term mental health after hurricanes Katrina: a longitudinal study of low-income survivors. Soc Sci Med. (2015) 138:38–43. doi: 10.1016/j.socscimed.2015.05.037, PMID: 26046725

[65] Brosschot JF Gerin W Thayer JF. The perseverative cognition hypothesis: a review of worry, prolonged stress-related physiological activation, and health. J Psychosom Res. (2006) 60:113–24. doi: 10.1016/j.jpsychores.2005.06.074, PMID: 16439263

[66] Kubzansky LD Kawachi I Spiro A Weiss ST Vokonas PS Sparrow D. Is worrying bad for your heart? Circulation. (1997) 95:818–24. doi: 10.1161/01.CIR.95.4.818, PMID: 9054737

[67] Abramson D Stehling-Ariza T Garfield R Redlener I. Prevalence and predictors of mental health distress post-Katrina: findings from the Gulf coast child and family health study. Disaster Med Public Health Prep. (2008) 2:77–86. doi: 10.1097/DMP.0b013e318173a8e7, PMID: 18520693

[68] Dinan T. Projected increases in hurricane damage in the United States: the role of climate change and coastal development. Ecol Econ. (2017) 138:186–98. doi: 10.1016/j.ecolecon.2017.03.034

[69] Petkova EP Ebi KL Culp D Redlener I. Climate change and health on the U.S. Gulf Coast: public health adaptation is needed to address future risks. Int J Environ Res Public Health. (2015) 12:9342–56. doi: 10.3390/ijerph120809342, PMID: 26270669 PMC4555284

